# Message framing and counseling of parents on children’s physical activity – an experimental study

**DOI:** 10.1080/21642850.2018.1515018

**Published:** 2018-08-27

**Authors:** Olivier Drouin, Meredith Young, Nicholas King

**Affiliations:** a Department of Pediatrics, McGill University, Montreal, Canada; b Centre for Medical Education, McGill University, Montreal, Canada; c Department of Medicine, McGill University, Montreal, Canada; d Biomedical Ethics Unit, McGill University, Montreal, Canada; e Department of Epidemiology, Biostatistics, and Occupational Health, McGill University, Montreal, Canada

**Keywords:** Behavioral economics, communication, framing, physical activity

## Abstract

How messages are framed (gain or loss frame) modulate the effect of health information on physical activity level in adults. The role of framing of health information messages to parents about their child's physical activity is unknown.

Adult participants (parents) were randomized to see a video that either emphasized the benefits of physical activity (gain frame) or the risks of physical inactivity (loss frame) in children. The primary outcome was the change in the reported level of physical activity for their children between baseline and two-week follow-up.

92 individuals participated in the study and we obtained follow-up data for 48 participants (20 gain frame and 28 loss frame). Using a generalized linear model, we found that the frame presented to parents significantly influenced the trajectory of their child’s physical activity (*p* = 0.03), with the loss frame condition resulting in more favorable trajectory. Both the willingness to pay for organized physical activities and the perceived barriers to physical activity were similar between the two intervention groups.

The change in the reported level of activity of the child over a two-week period was significantly different whether parents were exposed to a loss frame or a gain frame video message.

## Introduction

Rising prevalence of sedentary behavior and obesity in children has amplified the need for effective preventive counseling by physicians. In Canada, only one third of youth engage in an average of 60 min of moderate-to-vigorous physical activity per day, as recommended by national guidelines (Tremblay, Carson, & Chaput, 2016). Physical activity counseling has been shown to be cost-effective and desirable by most health professionals (Cobiac, Vos, & Barendregt, 2009; Price, Desmond, Ruppert, & Stelzer, 1989). However, physicians often feel overwhelmed and ill-prepared by the task of obesity prevention, and report limited efficacy in inducing behavior change (Barlow & Dietz, 2002; Franc, Van Gerwen, Le Vaillant, Rosman, & Pelletier-Fleury, 2009). Indeed, limited information is available to healthcare providers about the most effective ways to promote physical activity to parents and children (Lipnowski, Leblanc, Canadian Paediatric Society, L, & Sports Medicine, 2012). The problem might lie in the assumption that, given enough information and opportunity, patients will always choose the best available option for their health (Kahneman, 2011; Thaler & Sunstein, 2008). This assumption, central to most behavior change efforts in medicine, is based on the expected utility model developed and used widely in the field of economics (Thaler & Sunstein, 2008).

Behavioral economics is one approach that has attempted to explain the apparent discrepancy between expected behavior and observed ‘real life’ behavior of individuals, including violations of predictions made by the expected utility model (Cawley, 2004; Kahneman, 2011; Thaler & Sunstein, 2008). Prospect theory is one element of behavioral economics showing that individuals respond differently when facing a choice with uncertain outcomes, depending on whether the choice is presented in terms of gains or losses (Kahneman & Tversky, 1979). Message framing effect occurs when individuals make different decisions based on whether the same information is presented, or ‘framed’, in terms of gains versus losses (Kahneman, 2011; Kahneman & Tversky, 1979). For example, physicians are more likely to recommend a treatment whose ‘one-month survival rate is 90%’ than one that has ‘a 10% mortality in the first month’, despite the fact that these options are factually identical (McNeil, Pauker, Sox, & Tversky, 1982). Since the original description of prospect theory, researchers have explored the application of message framing to health behaviors, where the risks and benefits are often not immediate, and therefore uncertain (Abhyankar, O'Connor, & Lawton, 2008; Banks et al., 1995; Jones, Sinclair, & Courneya, 2003; Latimer et al., 2008). There has also been growing interest in using gain/loss framing techniques to promote socially desirable behaviors, including health prevention interventions (Gallagher & Updegraff, 2012; Keller & Lehmann, 2008). So far, studies have showed mixed results, with some finding larger benefits from loss- or gain-framed messages, and others finding no effect of framing (Arora, Stoner, & Arora, 2006; Bassett-Gunter, Stone, Jarvis, & Latimer-Cheung, 2017; Jones, Sinclair, Rhodes, & Courneya, 2004; Latimer, Brawley, & Bassett, 2010).

At present, however, it is still unclear how findings from message framing and prospect theory articulate with other behavior change models such as the theory of planned behavior, and whether constructs such as perceived value and barriers could mediate the effect of message framing. Interestingly, one meta-analysis reported that while gain frame messages does not result in statistically significant increases in attitude or intention to change, it does increase disease prevention behavior (Gallagher & Updegraff, 2012), suggesting that the effect of the information framing on behavior might not be mediated by behavior intention (Glanz, Lewis, & Rimer, 1990).

Preliminary investigations of the use of message framing to promote physical activity have shown promising results (Arora et al., 2006; Jones et al., 2004; Latimer et al., 2008). However, few studies have investigated the impact of framing to promote physical activity for children. The presence of a surrogate decision-maker (the parent or caregiver) as the focus of the framing intervention could influence the effect of framing in ways that are difficult to predict. To date, the majority of studies of health information framing in pediatrics have been on single events (e.g. vaccination, level of care in premature neonates) rather than longer-term or recurrent actions such as physical activity, which could lead to different results (Haward, Murphy, & Lorenz, 2008; Hendrix et al., 2014). One study recently examined the effect of message framing on motivating parental support for physical activity and found that there was no significant effect of framing on parental physical activity support behavior, with both gain- and loss-frame messages equally positively impacting parent support for physical activity (Bassett-Gunter et al., 2017).

In this study, it was hypothesized that the frame of the information provided to parents about physical activity (gain vs. loss frame) would impact the level of physical activity of their children. Specifically, this study tested the hypothesis that the change in physical activity between baseline and follow-up would differ between the two groups.

## Methods

### Participants and recruitment

The population for this study was drawn from parents attending scheduled general pediatrics appointments at the Montreal Children’s Hospital, affiliated with the McGill University Health Centre (Montreal, Canada) between December 2014 and April 2015. Participants had to be comfortable with French or English, and have a child aged 2–15 years old. Children who were overweight or obese were not specifically included or excluded, as an attempt to recruit a naturalistic sample of those attending the clinic. However, none of the clinics that children attended specifically addressed issues of weight or obesity. Parents were asked: ‘Do you believe that your child has limitation that prevents him/her from participating in physical activity?’. Those who answered positively were excluded from the study. There were no other exclusion criteria.

### Study design

This study used a two-arm randomized behavioral study design in which parents were randomly assigned 1:1 to one of two intervention arms.

### Procedure

Parents coming for a medical appointment were handed information about the study at check-in by the clinic administrative staff and directed to a research assistant for further information. It was made clear to prospective participants that the clinical staff and the research team were independent, and that decision to participate (or not) in the study would have no influence on medical care. After obtaining informed consent, a research assistant collected participants’ demographic information, covariates (factors described in the literature as influencing physical activity in children – see below), and baseline physical activity using the two-day Physical Activity Questionnaire (see below)(Burdette, Whitaker, & Daniels, 2004). Participants were then randomized to viewing one of the two versions of the video. Group allocation was determined using a random number list developed by one of the authors (OD) and generated using Microsoft Excel for Mac 2011, version 14. Immediately after viewing the video, participants were asked to fill in ‘willingness-to-pay’ and ‘barriers to physical activity’ questionnaires described below. For this first phase of the study, participants received a 15$ gift card and were invited to participate in a follow-up two weeks later. The analysis presented here includes only the participants who agreed to follow-up. The follow-up included the two-day Physical Activity Questionnaire, delivered via email or phone at the participant’s preference. Upon completion, participants received another 15$ gift card and a debriefing form about the study.

Details of the intervention, outcomes, and covariates are described below.

### Intervention

The intervention consisted of a 2-min video depicting one of the authors (OD), identified as a physician, discussing either the benefits of physical activity (gain frame) or consequences of physical inactivity (loss frame) among children (script of each video available on demand). The information in either video was identical except for the message framing. For example the gain frame video included sentences such as: ‘The risk of cancer is lower in active people. Active children become active adults who live longer and healthier’., While the loss frame video instead used the following formulation: ‘The risk of cancer is greater in inactive people. Inactive children become inactive adults who can be sicker and live shorter lives’.. The messages in the videos were developed by the authors, based on existing examples of message framing in the literature (Arora et al., 2006; McCall & Martin Ginis, 2004; van ‘t Riet, Ruiter, Werrij, & de Vries, 2010). No participant saw both videos.

### Outcomes

Our primary outcome was the difference in the level of physical activity of children between baseline and two-weeks follow-up. The parent-reported physical activity level of the child was measured at both time points using a two-day Physical Activity Questionnaire adapted from the literature (Burdette et al., 2004; Sallis, Prochaska, & Taylor, 2000). This tool had previously been used in a pediatric population and was validated against physical activity as measured by the accelerometer (Burdette et al., 2004). The two-day Physical Activity Questionnaire ask parents to report their child’s physical activity for two days (one weekday and one weekend day), for three periods of the day (morning, afternoon and evening). For each period, parents report the duration of physical activity of their child, choosing among one of five categories: 0, 1–15, 16–30, 31–60, or >60 min. Those categories are then coded 0–4 for each period, and summed for a maximum score of 12 (Burdette et al., 2004). Scores obtained over two days were averaged. This previously published tool was used, rather than requesting an absolute number of minutes, in order to facilitate parental recall and physical activity report (Burdette et al., 2004; Sallis et al., 2000).

Secondary outcomes included two indirect measures of physical activity (outdoor play and play in a park) included in the original version of the Physical Activity Questionnaire, that were coded following a coding procedure identical to that of the primary outcome. Those indirect measures were collected at baseline only. We also collected measures of willingness to pay and perceived barriers to physical activity as described below.

#### Willingness to pay

Participants were asked to complete a questionnaire on how much they would be willing to pay, in Canadian Dollars, for eight different hypothetical physical activity opportunities for their child (a measure of the perceived value of physical activity), including swimming lessons, access to a local beach or provincial park, etc. Examining the perceived value of a good by how much people are willing to pay for it is a previously used measure of the effect of framing in the behavioral economics literature (Yang, Vosgerau, & Loewenstein, 2013). To prevent an anchoring effect, no specific price was suggested (Kahneman, 2011; Tversky & Kahneman, 1974). Parents had the option to indicate their belief that the activity was not appropriate/applicable to their child.

#### Barriers to physical activity

Participants were presented with a list of potential barriers to physical activity adapted from the current literature such as time in parent’s schedule, child’s motivation, etc. (Council on Sports, Fitness, & Council on School, 2006; Lipnowski et al., 2012). Similarly to previous publications in framing and barriers to physical activity (McCall & Martin Ginis, 2004), participants were asked to rate on a 4-point descriptive Likert scale (not a barrier, slight, moderate, major barrier) how important they perceived the barrier to be in preventing more physical activity in their child. Barriers were subsequently dichotomized into minimal (not a barrier or slight barrier) vs. significant (moderate or major barrier) for analysis.

### Covariates

The research assistant also collected demographic information as well as information on factors described in the literature as influencing physical activity in children (screen time, parental level of physical activity, active/passive commute to school, etc.) (Lipnowski et al., 2012).

### Data analysis

The analysis was designed to evaluate the possibility that exposure to one message frame led to greater increase in the reported level of physical activity from baseline than exposure to the other frame. Given the paucity of similar studies and lack of pilot data, the target sample size was chosen to be of similar magnitude to studies published in the field of message framing and physical activity in adults (Gallagher & Updegraff, 2012).

To determine whether the intervention affected reported physical activity level at the two-week follow-up, generalized estimating equations (GEEs), were used an extension of the generalized linear model for regressions with repeated outcomes that allows for correlated outcomes (Fitzmaurice, Laird, & Ware, 2012). GEE with unstructured variance-covariance matrix was used to predict change in physical activity over time as a function of message frame while allowing for controlling for possible confounders. The main analysis was conducted ‘per protocol’ using data only from participants for whom both baseline and follow-up physical activity data were available. An ‘intent to treat analysis’ was also conducted, imputing follow-up physical activity data using a ‘last data carried forward’ approach (i.e. assuming no change in physical activity between baseline and follow-up). To evaluate the impact of framing on a willingness to pay for physical activity (a measure of perceived value) and perceived barriers to physical activity (a measure of perceived self-efficacy), Wilcoxon rank sum test and chi-square trend test were used respectively.

All analyzes were performed using SAS University edition. Results of the trial are reported following the Consolidated Standards of Reporting Trials (CONSORT) (see Appendix  1 for CONSORT checklist). The complete protocol of this study is available from the authors on demand. The study was approved by the Ethics Review Board of the McGill University Health Centre (protocol 13-416-PSY) on 19 August 2014. The funding sources had no role in the study.

## Results

### Participants

**Figure 1.**
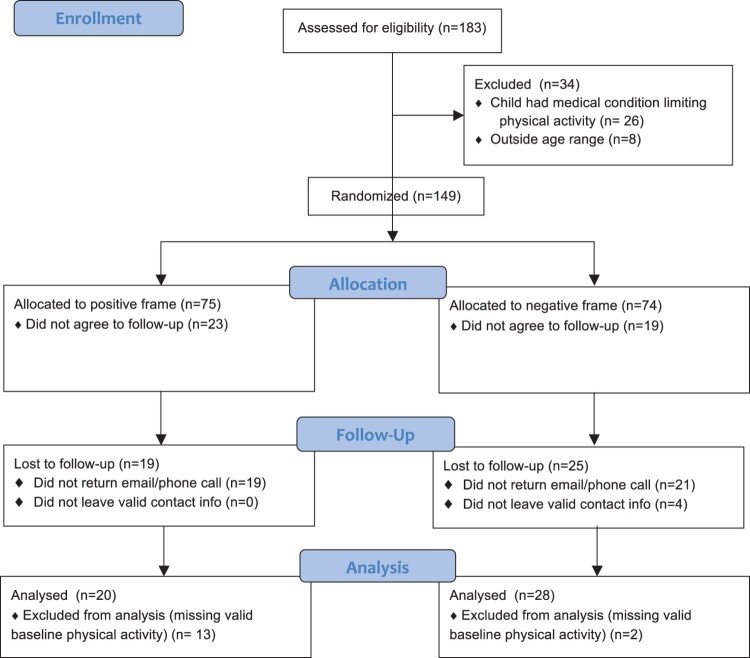
CONSORT flow diagram.

A total of 92 individuals participants consented to participate in both parts of this study: 39 viewed a gain frame video and 53 viewed a loss frame video. Follow-up data was available for 48 of those participants: 20 who had viewed the gain frame and 28 who had viewed the loss frame (see CONSORT Diagram – Figure 1).

#### Demographic data and baseline physical activity

Demographic data for the 48 participants and their children for whom follow-up data were available are presented in Table 1. Consistent with randomization, there was no statistically significant difference in the demographics of the two arms of the study. Participants were more likely to be women (87.5%), have a university-level education (60.4%) and report a family income of more than 75 000$/year (60.9%). Twenty-six percent of participants self-identified as part of a visible minority. The children of the participants had a mean age of 10.2 years, 54% of whom were girls. There were no statistically significant difference between the two experimental groups with regards to baseline physical activity of participants’ children, the reported time spent playing either outside or around the house, two other indirect physical activity measures (Table 1). There was also no statistically significant difference between those randomly assigned to the two video groups in variables previously described as linked to children’s level of physical activity (Lipnowski et al., 2012) except for the parental level of physical activity (Appendix  2).

**Table 1.** Respondent baseline sociodemographic information and physical activity predictors as distributed across message frames.

	Gain frame	Loss frame	*p*-value
	(*n* = 20)	(*n* = 28)	
*Participants (adults)*			
Mean age parent (years)	38.7	40.2	0.47
% female parent	90	85.7	1.00
% visible minority	26.3	26.9	1.00
Level of education (%)			0.34
Less than high school	0	3.6	
High school completed	20	7.1	
Some post-secondary education	15	32.1	
College graduate	25	35.7	
Some graduate school	40	21.4	
Income (%)			0.37
<25K$/yr	10	3.8	
25–50K$/yr	25	11.5	
50–75K$/yr	5	23.1	
75–100K$/yr	25	26.9	
>100K$/yr	35	34.6	
*Children of participants*			
Mean age of child (range)	10.3 (2.5–15)	10.1 (2–15)	0.88
% Female	50	42.9	0.77
Baseline level of physical activity (score out of 12)	5.1	4.4	0.33
Baseline level of outdoor play (score out of 12)	2.7	2.5	0.78
Baseline level of play in a park (score out of 12)	3.6	3.3	0.69

### Follow-up and impact of information frame

Distribution of physical activity at follow-up and children’s individual trajectory during the study period are presented in Appendices 3 and 4. Generalized linear model confirmed that there was no statistically significant difference in the level of physical activity between the two groups at baseline (difference of −0.30 [95% C.I. −1.14; 0.54]). The model showed that there was a significant effect of time on the reported level of physical activity for the participants in the gain frame condition with a decrease of 1.19 points on a 12-point scale over the two weeks period ([95% C.I. −2.16; −0.22] *p* = 0.02), but no statistically significant change for participants in the loss frame condition (+0.26 [95% C.I. −0.57; 1.09] *p* = 0.54) (Table 2). The model also demonstrated a significant interaction between the message frame and time (*p* = 0.03). Stated otherwise, among the 48 participants for whom follow-up data was available, the frame in which the message was presented to parents significantly influenced the trajectory of their child’s level of physical activity. This interaction remained significant even after adjusting for child’s age and gender, family income and parental level of education (Table 2).

**Table 2.** Generalized estimating equations regression results on the effect of message frame on child's physical activity across time (reference = Gain frame).

	Unadjusted model (AIC: 826.5)			Adjusted model (AIC: 691.9)		
	Effect estimate	95% C.I.	*p*-value	Effect estimate	95% C.I.	*p*-value
Mean PA level at baseline (gain frame)	5.06	(4.44; 5.67)		5.28	(4.18; 6.39)	
Mean change in PA (gain frame)	−1.19	(−2.16; −0.22)	0.02	−1.22	(−2.24; −0.20)	0.02
Mean PA level at baseline (loss frame)	4.76	(4.19; 5.33)		3.10	(−1.56; 7.77)	
Mean change in PA (loss frame)	0.26	(−0.57; 1.09)	0.54	0.29	(−0.62; 1.19)	0.53
Difference in PA at baseline (Ref = gain frame)	−0.30	(−1.14; 0.54)	0.48	−0.08	(−1.00; 0.84)	0.84
Frame x time interaction (Ref = gain frame)	1.45	(0.17; 2.72)	0.03	1.51	(0.14; 2.88)	0.03
Covariates						
Income category						0.75
<25K$/yr				REF		
25–50K$/yr				−0.28	(−2.21; 1.65)	
50–75K$/yr				−0.90	(2.79; 0.99)	
75–100K$/yr				−0.58	(−2.48; 1.33)	
>100K$/yr				−0.19	(−2.00; 1.62)	
Level of education						0.09
Less than high school				REF		
High school completed				3.22	(−0.95; 7.40)	
Some post-secondary education				3.27	(−0.84; 7.38)	
College graduate				1.93	(−2.18; 6.04)	
Some graduate school				2.26	(−1.91; 6.44)	
Gender of the child (REF = boys)				−0.27	(−1.12; 0.57)	0.52
Age of the child				−0.01	(−0.12; 0.09)	0.80

Notes: Adjusted model: adjusted for income, parent's educational achievement, child gender, and child age. AIC: Akaike information criterion, C.I.: confidence interval; PA: physical activity.

Using an intent-to-treat analysis and imputing follow-up data when required, the relationship described above remained, with a statistically significant interaction term between message frame and time, both in the unadjusted, and adjusted analyses (Appendix 5).

There was no significant difference in perceived value of physical activity (as measured by willingness to pay for physical activity) between the gain-frame and loss-frame conditions (Appendix 6). Similarly, except for the perceived lack of information, there was no statistically significant difference between the two conditions in the percentage of participants reporting that the proposed barriers were important (Appendix 7).

## Discussion

To our knowledge, this is the first randomized study investigating the use of health information framing for physical activity behavior in children. A single short exposure to a gain- vs. loss-frame video message led to a significant difference in the trajectory of reported physical activity. Message framing did not appear to influence the perceived value of physical activity (willingness to pay) or participants self-efficacy (perceived barriers), a finding consistent with a meta-analysis on message framing in health promotion interventions (Gallagher & Updegraff, 2012).

The results of this study contrast with studies in adults where gain frame messages seemed to be more effective in increasing physical activity (Latimer et al., 2008; McCall & Martin Ginis, 2004). The difference in medium (use of a video in this case versus written message), as well as the presence of a surrogate decision-maker (the parent) may contribute to the difference in our findings compared to those in the literature (Latimer et al., 2008). For example, a recent study on the use of message framing on parental support for their child’s physical activity showed no effect of message framing (Bassett-Gunter et al., 2017). In addition, among the few studies examined the effect of framing on health information in a video medium (arguably closer to mimicking the ‘live’ health information encounters than written study materials) most, but not all, found a larger effect for the loss frame messages (Banks et al., 1995; Rivers, Salovey, Pizarro, Pizarro, & Schneider, 2005; Schneider et al., 2001). Another possible contributor is that risk aversion is thought to be greater when adults take decisions for the health of their children than when they take similar decisions regarding their own health, a situation that could modulate the effect of message framing (Hammitt & Haninger, 2010; Kahneman, 2011; Kahneman & Tversky, 1979).

Limitations of this study include the use of recall questionnaires as a measure of physical activity for another person (the child). However, potential recall bias should not have been different between randomized groups with similar demographic profiles. In addition, while there is an inherent error in the use of recall questionnaires, an attempt was made to minimize its potential impact by adapting a tool previously developed and used in pediatrics (Burdette et al., 2004). The original tool was however validated against objective accelerometer data only in a segment of our study population (preschool children) and for a subset of physical activity (outdoor play). With regards to the intervention, our study only included a loss- and a gain-framed message, but not a no-message, or control message (about a different topic for example) condition. We made this choice to more closely reflect clinical practice, but in doing so, we cannot infer what would have been the physical activity trajectory of such a control group. Despite the relatively short period between the intervention and follow-up, loss to follow-up was apparent, despite our efforts to contact participants following the two-week delay. There was no difference in the collected baseline characteristics, including reported baseline level of physical activity and known predictors of physical activity, between participants who followed-up and those who did not (Appendix 8). There are other factors influencing physical activity that could have been collected, but we limited them to limit the burden on the participants. In addition, given the randomized nature of our study, it is likely that those unmeasured factors would have been balanced between both groups. The sample size led to insufficient power to perform subgroup analysis to evaluate if the impact of the intervention was more or less effective in participants with certain demographics, including certain age groups, but the effect remained significant after adjusting for basic demographics. It is also possible that the small sample size, and the lack of a formal sample size calculation, led to insufficient power to detect small effect sizes. Generalizability of this study is limited by the fact that while recruitment was carried out among all eligible participants, participants demographics were skewed towards participants who were women and of higher socioeconomic status. Recruitment was also performed in a university health center, where the patient population may be different from a community practice. This difference was hopefully limited by recruiting participants from parents in the general pediatrics clinic of a Canadian hospital that provides publically accessible health care, and by excluding from our analysis data about children who had a health condition that could limit their capacity to physical activity.

## Conclusion

This study shows that small differences in the presentation of health information can lead to a statistically significant difference in reported health behaviors. Simple changes in how information is communicated to parents could help clinicians encourage families to increase the amount of physical activity done by their children, and so should be considered as a simple and achievable recommendation for clinical practice.

## Supplementary Material

Appendix_8.xlsx

Appendix_7.xlsx

Appendix_6.xlsx

Appendix_5.xlsx

Appendix_4.pdf

Appendix_3.xlsx

Appendix_2.xlsx

Appendix_1CONSORT_Checklist.doc
