# Supplementary figures and images for: Message framing and counseling of parents on children’s physical activity – an experimental study
Source: Health Psychol Behav Med. 2018 Aug 27;6(1):214–25. doi: 10.1080/21642850.2018.1515018 (PMC8114350; doi:10.1080/21642850.2018.1515018)

Spaghetti plot - Positive frame

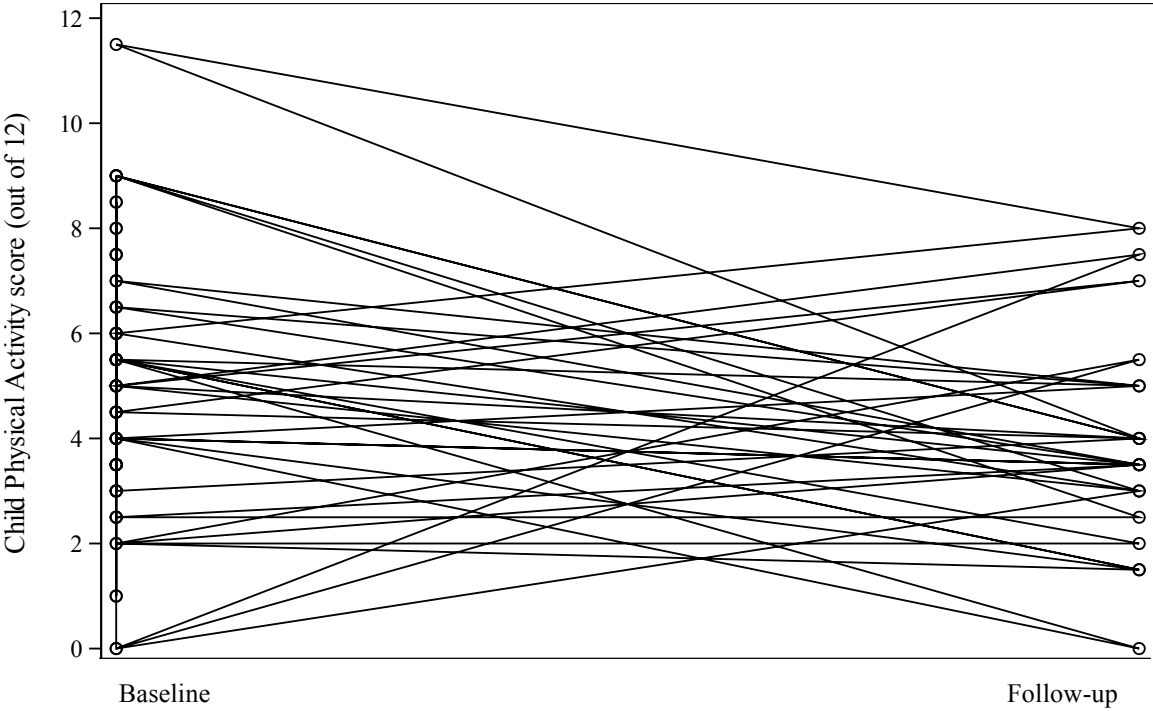

Spaghetti plot - Negative frame

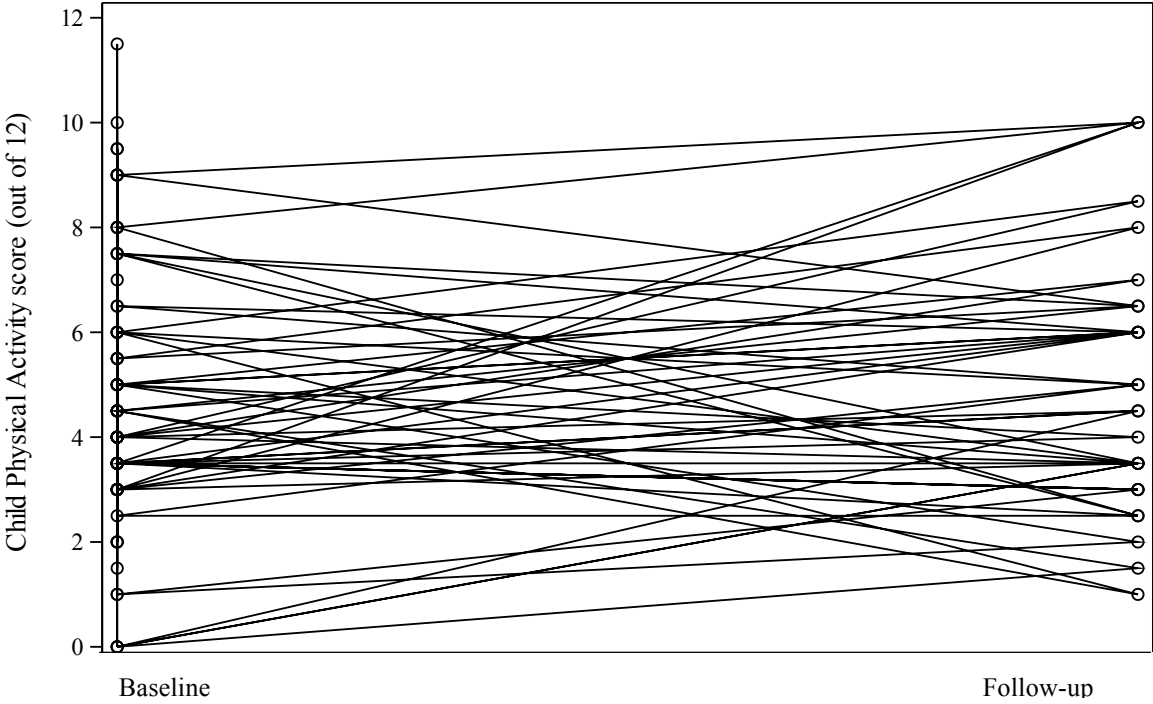

Supplement: Appendix_4.pdf [file RHPB_A_1515018_SM3922.pdf]
